# Cost-effectiveness analysis of vagus nerve stimulation in drug-resistant epilepsy

**DOI:** 10.1186/s42494-025-00219-1

**Published:** 2025-11-01

**Authors:** Weixi Xiong, Lu Lu, Yingying Zhang, Caleb Onyenaturuchi Egbuta, Xintong Wu, Josemir W. Sander, Dong Zhou

**Affiliations:** 1 Department of Neurology, West China Hospital of Sichuan University, Chengdu, Sichuan 610041 China; 2 NIHR University College London Hospitals Biomedical Research Centre, UCL Queen Square Institute of Neurology, Queen Square, London, WC1N 3BG UK; 3 Chalfont Centre for Epilepsy, Chalfont St Peter, UK

**Keywords:** Seizure, Refractory epilepsy, Neurostimulation, Health economics

## Abstract

Epilepsy is a chronic neurological disorder characterized by recurrent, unprovoked seizures that affect approximately 50 million people worldwide. Despite the availability of numerous anti-seizure medicines (ASMs), about 30% of people develop drug-resistant epilepsy (DRE), defined as failure to achieve sustained seizure freedom after trials of two appropriately chosen and tolerated ASM regimens. This population faces significantly reduced quality of life, increased mortality risks, and substantial socioeconomic burdens due to frequent hospitalizations and limited employability.

For these treatment-resistant cases, neurostimulation therapies have emerged as promising alternatives to conventional pharmacotherapy. Among these, vagus nerve stimulation (VNS) has become one of the most widely used neurostimulation techniques since approved in 1997. Clinical studies demonstrated that VNS provides meaningful clinical benefits, with approximately 50–60% of patients achieving over 50% reduction in seizure frequency within 12–24 months after implantation. Beyond seizure control, VNS has been associated with improved mood, cognition, and quality of life measures. The therapy is particularly valuable for patients not candidates for resective surgery.

This paper presents a comprehensive cost-effectiveness analysis of VNS in DRE by reviewing relevant literature. We examine three key economic dimensions: (1) direct medical costs (including device implantation and maintenance), (2) indirect societal costs (such as productivity loss), and (3) long-term economic benefits. Our analysis reveals that in published papers mostly from developed countries, while VNS requires initial investment, it demonstrated remarkable long-term cost-effectiveness. The therapy significantly reduces healthcare utilization, medication costs, and socioeconomic burdens associated with uncontrolled epilepsy. Furthermore, we identify critical factors influencing cost-effectiveness and propose evidence-based optimization strategies to enhance the value proposition of VNS therapy for diverse healthcare systems and selected patients.

## Background

Epilepsy is a common, chronic neurological disorder that imposes a substantial economic burden on patients and society. Anti-seizure medications (ASMs) are the first-line treatment for epilepsy. However, 20–30% of epilepsy patients have drug-resistant epilepsy (DRE), where seizures cannot be controlled by medications. After a detailed preoperative evaluation, some drug-resistant cases may achieve better clinical outcomes through resective surgery. Unfortunately, a significant proportion of patients are not suitable for surgery or are unwilling to undergo craniotomy.

Vagus nerve stimulation is a therapeutic approach that modulates brain activity through electrical stimulation of the vagus nerve. Since its approval by the FDA in 1997 for treating drug-resistant epilepsy, this technology has been widely adopted globally [1]. The VNS system consists of an implantable pulse generator, leads, and electrodes, which are surgically implanted in the patient's body to deliver periodic electrical stimulation for seizure control.

As an innovative neuromodulation therapy, VNS provides a new treatment option for patients with refractory epilepsy. Clinical studies have shown that VNS can significantly reduce seizure frequency and improve patients' quality of life. However, VNS therapy also has limitations, including the need for long-term device implantation, potential side effects such as hoarseness, and relatively high treatment costs. These factors must be incorporated when evaluating the cost-effectiveness of VNS.

Compared to other epilepsy treatment options, the high cost of VNS has raised widespread concerns about its cost-effectiveness. The American Academy of Neurology has stated: "Vagus nerve stimulation devices and implantation procedures are expensive. This approach is cost-effective only when studies demonstrate reductions in physician and emergency department visits, decreased reliance on anti-epileptic drugs, and improved quality of life" [2]. However, due to differences in national healthcare systems and specific country contexts, the cost-effectiveness of VNS in China remains unclear.

This article aims to review relevant literature, thoroughly examine the direct and indirect costs of VNS, assess its long-term economic benefits, and analyze key factors affecting cost-effectiveness, thereby providing recommendations for optimizing the clinical application of VNS in China.

## Methods

### Search strategy

We systematically searched authoritative databases such as PubMed, Web of Science, Embase, CNKI, and Wanfang Data. Key search terms included "vagus nerve stimulation," "refractory epilepsy," "cost-effectiveness analysis," "cost-utility analysis," and "cost-benefit analysis," combined with Boolean operators to construct search queries. For example, in PubMed, the query was structured as follows: "(vagus nerve stimulation AND refractory epilepsy) AND (cost-effectiveness OR cost-utility OR cost-benefit)."

### Inclusion criteria

Included studies were original research, systematic reviews, or meta-analyses related to the cost-effectiveness of VNS for epilepsy. We limited the study population to patients with refractory epilepsy who received VNS therapy. We excluded animal studies, non-cost-effectiveness analyses, and duplicate publications. Through screening, we retained high-quality and highly relevant literature for subsequent analysis.

## Results

### Cost of vagus nerve stimulation therapy

The cost analysis of VNS involves direct and indirect medical costs. Direct costs primarily consist of device expenses, surgical fees, and postoperative follow-up and maintenance costs. The VNS device itself is costly, and when combined with surgical and hospitalization fees, the initial treatment cost is significantly higher compared to medication. After implantation, patients require regular follow-ups and device maintenance, which contribute to sustained healthcare spending over time.

This cost structure highlights the high upfront investment required for VNS, along with ongoing expenses that must be considered in economic evaluations.

### Cost-effectiveness analysis of vagus nerve stimulation therapy

#### Direct medical cost

##### Device and surgery-related costs

The VNS device (including the pulse generator and electrodes) is costly, and implantation requires specialized medical teams and equipment, resulting in high surgical fees. These costs vary significantly between countries. In China, imported devices are typically more expensive than domestic ones, increasing upfront patient investments. Preoperative evaluations, diagnostic tests, postoperative programming, follow-ups, battery replacements, and device maintenance also incur additional expenses. A U.S. study found that outpatient visits accounted for over 50% of total medical costs after VNS implantation [3].

##### Medication costs

Most patients require ASM treatment after the VNS implantation. Whether the number of medications decreases after VNS remains debated, but most studies suggest a reduction in ASM costs. A prospective Chinese study of 61 patients found no significant reduction in the number of ASM types at the 18-month follow-up [4]. Another small U.S. prospective cohort study reported that 43% of patients reduced their numbers of ASM [5].

##### Complications and device removal costs

VNS complication rates are low (3–6%), primarily including local infections and vocal cord paralysis, most of which can be managed conservatively. A small percentage of people require device removal, but the costs are far lower than severe complications from craniotomy (e.g., intracranial hemorrhage or neurological deficits). A retrospective study of 436 patients found that 17% underwent device removal after implantation, with an average removal time of 40 months from initial placement, and the reasons included inefficacy and complications [6].

#### Indirect medical cost and social benefits

Indirect costs vary greatly between countries and regions, as their calculation requires precise access to social policy data. Most studies do not include indirect costs. However, potential reductions in indirect costs from VNS may include:

##### Reductions in net healthcare costs

The healthcare utilization and cost burden for the VNS candidate is huge, especially when the implantation draws near. A study based on a United States healthcare claims database showed that the mean costs were $123,500 per person within the 2 years before implantation [7]. Multiple studies report that VNS implantation reduces seizure frequency, leading to economic benefits that offset some costs. These include decreased hospitalizations, outpatient visits, emergency care [8, 9], and reduced use of anti-seizure and rescue medications [10]. Studies also show that VNS reduces severe epilepsy-related events such as fractures, traumatic brain injuries, and status epilepticus [3].

##### Improvements in work capacity rehabilitation and daily productivity metrics

No long-term data are available to assess social outcomes, but some studies found no short-term improvement in employment rates or income levels post-VNS; in some cases, the number of disability pension recipients slightly increased [10]. Another study from the United States noted significant reductions in health-related absenteeism and time spent managing health issues [11]. Discrepancies in labor outcome studies may reflect that patients receiving VNS often already have severely impaired work capacity. Whether earlier VNS intervention could better reverse epilepsy's negative effects on productivity requires further research.

##### Reduced family caregiver burden

Before 2017, VNS was indicated for patients aged over 12 years [1]; since 2017, the indication expanded to age over four [12]. A prospective multicenter study from Taiwan province in China showed that VNS reduced seizure frequency in pediatric patients, thereby alleviating parental stress, especially in younger children [13].

#### Comparative cost-effectiveness of different treatment options

##### Comparison with anti-seizure medication

Multiple studies from Europe and the U.S. show that post-VNS medical costs were reduced compared to continued pharmacotherapy alone. The greater the cost difference between controlled and uncontrolled epilepsy in different regions/patient groups, the higher VNS's projected cost-effectiveness [14]. Two Belgium prospective cohort studies suggest VNS is cost-effective, compared to those continued with ASM, the cost of VNS was saved within two years following implantation [15, 16]. Another prospective study from Belgium, focused on whether VNS reduced epilepsy-related direct medical costs (ERDMC), which account for only 25–30% of total epilepsy-related costs in patients with refractory seizures, and thereby allowed total costs reductions. The results showed that in ineligible surgical candidates, compared to conservative management, VNS significantly reduced ERDMC, but it would take some years to balance the costs [17]. Studies and reports from the U.K. were aligned: a meta-analysis of VNS randomized trials found improved seizure control avoided £745 annually in healthcare costs [18]. A more recent U.K. study estimated an incremental cost-effectiveness ratio (ICER) of £17,771 per QALY (quality-adjusted life years) gained for VNS + ASM vs. ASM alone [8]. Longer follow-up (6 years) of a 704 patients’ real-world study also confirmed significant cost savings, mostly associated with a reduction of epilepsy-related hospitalization. While the clinical benefits were ambiguous, the financial outcomes were significantly improved [19]. Reports form the United States also indicated that VNS is highly cost-effective, especially in the US Medicare context, which could save $109,678 per patient and gain 0.385 QALYs compared to ASM alone [11]. In populations of different insurances, VNS was all found to be cost-neutral within 2 years of implantation [9, 11, 20]. However, the VNS cost-effectiveness in pediatrics remains debated, with fewer studies [21–26].

Fewer studies exist in developing countries but show similar trends. A small Jordanian survey found a 30% economic burden reduction per patient, though savings were lower than in high-income countries—likely due to lower baseline healthcare costs but similar VNS implantation expenses [27]. The studies are summarized in Table 1.

**Table 1:** Summary of Current Studies

Ref	Study type	Time	Nation	Population	Conclusion
[3]	Pre-post analysis of Medicaid data	January 1997 to June 2009	United States	*N* = 1655	VNS is associated with decreased net cost savings after 1.5 years
[8]	An Excel model developed according to NICE guidance	Published in 2021	United Kingdom	NA	VNS is expected to be a cost-effective intervention in the treatment of DRE
[9]	A retrospective, observational, cohort study of Medicare and Medicaid data	January 2011 to December 2020	United States	*N* = 16,223	VNS implantation was associated with 41% and 52% reductions in all-cause and epilepsy-related hospitalizations and ED visits
[11]	A cohort state transition model based on published clinical trials and registry data	Published in 2023	United States	NA	VNS was cost effective in the US Medicare context
[16]	Perspective cohort study	Publied in 1999	Belgium	*N* = 15	VNS is an efficacious and cost-beneficial treatment for refractory partial seizures
[17]	Perspective cohort study	Publied in 2002	Belgium	*N* = 84	VNS reduced the epilepsy-related direct medical costs
[18]	Retrospective cohort study	Publied in 2003	United Kingdom	*N* = 42	No strong economic argument against VNS implantation
[19]	Retrospective longitudinal open-cohort	April 2008 to July 2014	England	*N* = 704	VNS reduced in long-term epilepsy-related medical costs
[20]	An Markov model	Publised in 2018	United States	NA	VNS had long-term lower resource utilization and costs
[24]	A Markov decision analytical model	Publised in 2015	Netherlands	NA	VNS is not cost effective options compared to CAU
[25]	Real world retrospective study of Medicaid data	Publised in 2012	United States	*N* = 238, aged 1–11 years	VNS in pediatric patients is associated with decreased healthcare resource use and cost
[26]	Retrospective cohort study	January 2011 to December 2016	United States	*N* = 1113, aged under 18	VNS is cost-beneficial treatment in pediatric patients with refractory epilepsy
[27]	Retrospective review	2007 to 2011	Jordan	*N* = 28, aged under 18	QALY gain and cost per QALY analysis were encouraging
[28]	A Markov decision analytic model	Publised in 2022	Netherlands	NA	VNS and DBS are potentially cost-effective compared to CAU
[29]	Retrospective of healthcare claim database	2012 to 2019	United States	*N* = 792	All-cause and epilepsy-related costs was statistically significantly lower for VNS compared to DBS/RNS

*VNS* Vagus nerve stimulation, *NA* Not applicable, *ED* Emergency department, *CAU* Care as ususal, *QALY* Quality-adjusted life years, *DBS* Deep brain stimulation, *RNS* Responsive neurostimulation

##### Comparison with non-pharmacologic therapies

Direct comparison between resective surgery and VNS implantation is challenging as people receiving VNS were normally ineligible surgical candidates. VNS has higher initial costs but lower complication/rehabilitation expenses (Table 1). One study of children with Lennox-Gastaut syndrome found VNS was more cost-effective than corpus callosotomy, despite the latter having superior clinical outcomes, especially for atonic seizures [22]. When comparing to ketogenic diet (KD), a high-fat, low-carbohydrate therapeutic diet that mimics fasting metabolism to induce ketosis, primarily used as an alternative treatment for DRE, a pediatric study from the Netherlands found KD cheaper and more effective than VNS in the first 12 months, but by year five, KD remained cheaper though less effective [24]. Comparative cost-effectiveness studies of different neurostimulation approaches for epilepsy remain particularly scarce, one study noted deep brain stimulation (DBS) had marginally better incremental cost-per-QALY than VNS, though DBS's efficacy uncertainty warrants further study [28]. A study based on U.S. insurance claims databases found that people who had VNS implantation had significantly lower 24-month post-implant healthcare costs (including all-cause and epilepsy-related) than those had responsive neurostimulation system/DBS, even excluding intracranial device placement fees [29].

### Cost-effectiveness analyses in specific groups

Although studies of VNS in specific subgroups of epilepsy had been reported, few focused on cost-effectiveness (see Table 2).

**Table 2:** Cost-effectiveness analyses in specific groups

Patients	Study type	Time	Nation	Conclusion
Drug-resistant epilepsy in children with tuberous sclerosis sclerosis [21]	Review	1990–2015	NA	Comparing introducing the fourth ASM and resective epilepsy surgery, VNS is more expensive but less effective, so not prioritized
Lennox-Gastaut syndrome [23]	Cohort study	Before 2001	Netherlands	Compared to 6 months before VNS, the costs during the 6 postoperative months are 2,876.06 Euros less; the payback period is 2.3 years
Lennox-Gastaut syndrome [22]	Analysis based on current literature	Before 2021	NA	CC had a 15% greater likelihood of a positive seizure outcome, but per positive seizure outcome gained was 451,952 U.S dollars more than VNS

*NA* Not applicable, *ASM* Anti-seizure medicine, *VNS* Vagus nerve stimulation, *CC* Corpus callosotomy

### Limitations

The cost-effectiveness of neuromodulation therapies exhibits substantial variation across different patient populations, reflecting inherent clinical and biological heterogeneity. Pediatric epilepsy patients often demonstrate distinct response patterns compared to adults, while those with generalized epilepsy syndromes like Lennox-Gastaut typically require more intensive stimulation protocols and frequent device adjustments than their focal epilepsy counterparts. These differential requirements directly impact long-term expenditures — for instance, the increased energy demands of higher stimulation parameters in refractory generalized epilepsy cases lead to more frequent generator replacements, substantially altering the cost-benefit calculus. Current economic evaluations remain constrained by relatively short follow-up periods, with most available data spanning 5–10 years post-implantation. This temporal limitation obscures a critical understanding of ultra-long-term (>20 years) economic impacts, particularly regarding multiple device replacement cycles in younger patients and the cumulative quality-of-life benefits that may accrue over decades. The field urgently requires prospective, longitudinal studies employing standardized outcome measures to capture these dynamic cost-effectiveness relationships across the full patient lifespan and spectrum of epilepsy subtypes. Such data would enable more nuanced value assessments that account for both clinical heterogeneity and the evolving nature of neurostimulation costs and benefits over time.

### Future research directions

The advancement of vagus nerve stimulation therapy necessitates multidimensional optimization strategies to enhance its economic viability. A critical priority involves developing precision cost modeling frameworks that dynamically incorporate clinical variables including seizure classification profiles, pharmacological response patterns, and comorbid disease burdens to generate patient-specific cost-effectiveness predictions. Concurrently, healthcare policy reforms should prioritize the integration of VNS into national insurance formularies while implementing value-based reimbursement mechanisms such as performance-linked payment models, thereby alleviating financial barriers for eligible patients. From a technological perspective, innovation efforts should focus on engineering rechargeable pulse generators and perfecting transcutaneous VNS (tVNS) delivery systems to minimize surgical interventions and extend device longevity. Economic modeling suggests particular promise in battery life extension—Swedish healthcare data demonstrate annual savings of approximately $3,000 per patient through reduced emergency hospitalizations, with projections indicating that achieving battery durability beyond 8 years could render cumulative savings equivalent to or surpassing initial device acquisition costs [30]. These synergistic approaches—combining predictive analytics, policy restructuring, and biomedical engineering—collectively address the major economic constraints currently limiting optimal VNS implementation.

To further improve the value proposition of VNS therapy, three key research priorities should be addressed: First, the refinement of patient selection criteria through predictive biomarkers could enhance responder rates. Second, standardized surgical protocols and follow-up care pathways may reduce complication rates (currently 3–6% for minor complications). Third, technological advancements such as rechargeable generators and improved battery longevity could significantly decrease lifetime treatment costs.

Notably, the existing cost-effectiveness models primarily are derived from data in North America and Western Europe, where high baseline costs for managing uncontrolled epilepsy (particularly frequent hospital admissions) amplify the relative savings from VNS therapy. The generalizability of these findings to China's distinct healthcare environment—with different cost structures for hospitalization, medication pricing, and workforce participation—requires rigorous verification. There is a particular need for:

- Prospective Chinese studies with over 5-year follow-up tracking both clinical and economic outcomes
- Real-world evidence from domestic epilepsy centers using standardized cost-accounting methods
- Health technology assessments incorporating China-specific quality-of-life metrics

## Conclusions

Comprehensive analysis of available clinical and economic data indicates that VNS represents a cost-effective long-term therapy for drug-resistant epilepsy, despite substantial upfront costs associated with device implantation and surgical procedures. The economic benefits of VNS emerge progressively through multiple mechanisms: (1) sustained reduction in seizure frequency (typically 40–50% median reduction at 12–24 months), (2) decreased reliance on expensive anti-seizure medications, (3) reduced hospitalization rates and emergency care utilization, and (4) measurable improvements in patients' ability to maintain employment and daily activities. These combined effects typically offset the initial investment within 1.5–3 years in Western healthcare systems, with sustained benefits accruing over the device's 8–10 years lifespan.

Such locally-derived evidence will be essential for developing appropriate reimbursement policies and clinical guidelines that maximize the public health benefit of VNS therapy within China's healthcare system.

## Data Availability

Not Applicable.
